# A case report of postcardioversion device-related thrombus in a patient with left atrial appendage occlusion device on apixaban

**DOI:** 10.1097/MS9.0000000000001735

**Published:** 2024-01-22

**Authors:** Hasaan Ahmed, Mahmoud Ismayl, Anirudh Palicherla, Miranda Heppler, Terezia Petraskova, Omar Kousa, Jalal Vargha

**Affiliations:** a Department of Medicine, Division of Internal Medicine; b Department of Medicine, Division of Cardiovascular Disease, Creighton University School of Medicine, Omaha, Nebraska; c Department of Cardiovascular Medicine, Mayo Clinic School of Medicine, Rochester, Minnesota, USA

**Keywords:** atrial fibrillation, case-report, left atrial appendage occlusion device, thrombus, watchman

## Abstract

**Background:**

Current guidelines recommend proceeding with cardioversion, without the explicit need for preprocedural transesophageal echocardiography (TEE), in patients compliant with oral anticoagulation for at least 3 weeks. The relevance of these guidelines remains unclear in those undergoing repeat cardioversion.

**Case summary:**

A 66-year-old male with a history of atrial fibrillation (AF) and a left atrial appendage occlusion (LAAO) device, compliant with apixaban, presented with dyspnea and lightheadedness. He was cardioverted into sinus rhythm, 10 days before symptom onset, with TEE unremarkable at the time. An ECG revealed that the patient converted back into AF and a repeat cardioversion was scheduled. At the patient’s request, a TEE was obtained, revealing a new 2 cm×1 cm thrombus in the left atrium above the WATCHMAN device. Cardioversion was canceled and the patient was hospitalized for AF management.

**Discussion:**

Cardioverted patients are at risk for thrombus formation due to atrial stunning, a transitory dysfunction of the atrial appendage and atrium, which occurs immediately after cardioversion and can persist for several weeks. The likelihood of a thrombus is further propagated by individual risk factors for stroke.

**Conclusion:**

Anticoagulation does not eliminate the risk of thrombus formation in those with increased risk factors for stroke. Further studies are warranted to assess the need for routine TEE, after cardioversion, in those with stroke risk factors on anticoagulation or who have LAAO.

## Introduction

Highlights

- Current guidelines do not explicitly recommend obtaining a transesophageal echocardiogram (TEE) before cardioversion in atrial fibrillation patients compliant with anticoagulation for at least 3 weeks.
- Patients with atrial fibrillation are at risk of thrombus formation, after cardioversion, due to atrial stunning.
- The degree of atrial stunning can be appreciated by a TEE.
- The TEE remains the gold standard in identifying thrombi in the left atrial appendage.
- Routine surveillance TEE may be beneficial in recently cardioverted patients, with left atrial appendage occlusion devices, that have increased risk factors for stroke.

Atrial fibrillation (AF) remains the most prevalent type of cardiac arrhythmia and the leading cause of preventable ischemic stroke due to cardiac etiology^1^. While oral anticoagulation has been the mainstay of therapy in stroke prevention amongst patients with nonvalvular AF, left atrial appendage occlusion (LAAO) devices have risen in popularity, particularly amongst individuals who are not appropriate for long-term oral anticoagulation or who have contraindications to oral pharmacotherapy^1^.

Guidelines by both the American Heart Association/American College of Cardiology and the European Society of Cardiology on AF management recommend proceeding with cardioversion in patients on anticoagulation for at least 3 weeks, without the explicit need for preprocedural transesophageal echocardiography (TEE)^2,3^. However, there are no definitive guidelines with respect to direct current cardioversion (DCCV) in patients with LAAO devices^4^. Our case is unique in that the patient, despite being compliant with oral anticoagulation, was noted to have a significant thrombus on his LAAO device on TEE prior to cardioversion, despite a TEE 10 days earlier which showed no evidence of a thrombus. Our case, which has been reported in line with SCARE (Surgical Case REport) criteria, aims to evaluate and build upon current literature regarding the role of TEE in patients with LAAO devices compliant with anticoagulation undergoing repeat cardioversion^5^.

## Case presentation

**Figure 1:**
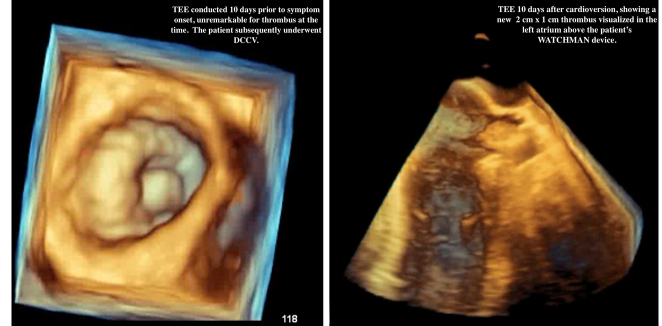
Transesophageal echocardiography images before cardioversion and after.

A 66-year-old caucasian male, with a past medical history of heart failure with preserved ejection fraction, symptomatic persistent AF with a LAAO device compliant on apixaban 5 MG BID, hypertension, stage 2 chronic kidney disease, and coronary artery disease, presented to the university cardiology clinic with fatigue, lightheadedness, and exertional dyspnea. The patient, a retired warehouse worker, had undergone DCCV 10 days prior to symptom onset for which sinus rhythm was restored. The TEE obtained before cardioversion showed a normal ejection fraction with no wall motion abnormalities and no thrombus present on the left atrial appendage (Fig. 1).

**Figure 2:**
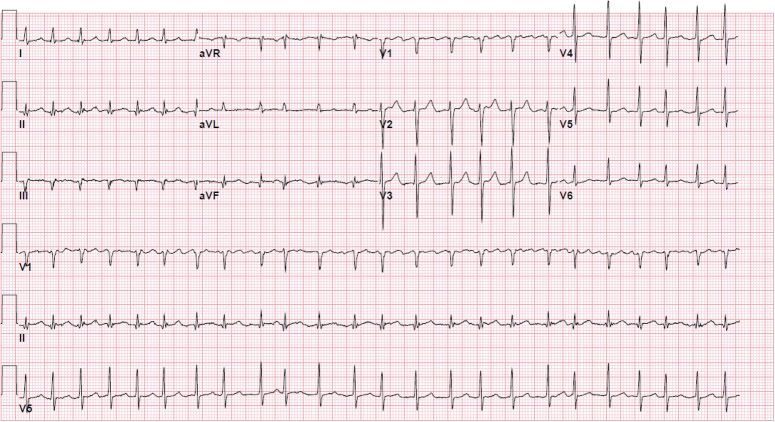
ECG notable for atrial fibrillation with rapid ventricular response.

At the clinic, the patient was found to have reverted back into AF with rapid ventricular response (Fig. 2) with physical exam remarkable for the patient being tachypenic, in moderate distress, and having an irregularly irregular pulse.

Of note, the patient had his WATCHMAN device implanted about 2 years prior to symptom onset, with a TEE showing successful placement of the device within the left atrial appendage and no thrombus or peridevice leak noted. Forty-five days after implantation, the patient underwent a TEE as part of a routine postwatchman device assessment, which again showed appropriate device positioning with no thrombus or peridevice leak noted.

Given that the patient had reverted back into AF, cardioversion was tenatively planned. At the request of the patient; however, a TEE was obtained prior to cardioversion, which incidentally found a 2 cm×1 cm thrombus visualized in the left atrium above the WATCHMAN device (Fig. 1). TEE also was notable for a normal ejection fraction and no wall motion abnormalities. The cardioversion was subsequently canceled and the patient was hospitalized for AF management. The labs obtained on admission were unremarkable. Digoxin was started for rate control and the patient’s home metoprolol was titrated up to 100 MG BID. The patient’s symptoms stabilized and he was discharged on warfarin with enoxaparin bridge and aspirin 81 mg daily.

**Figure 3:**
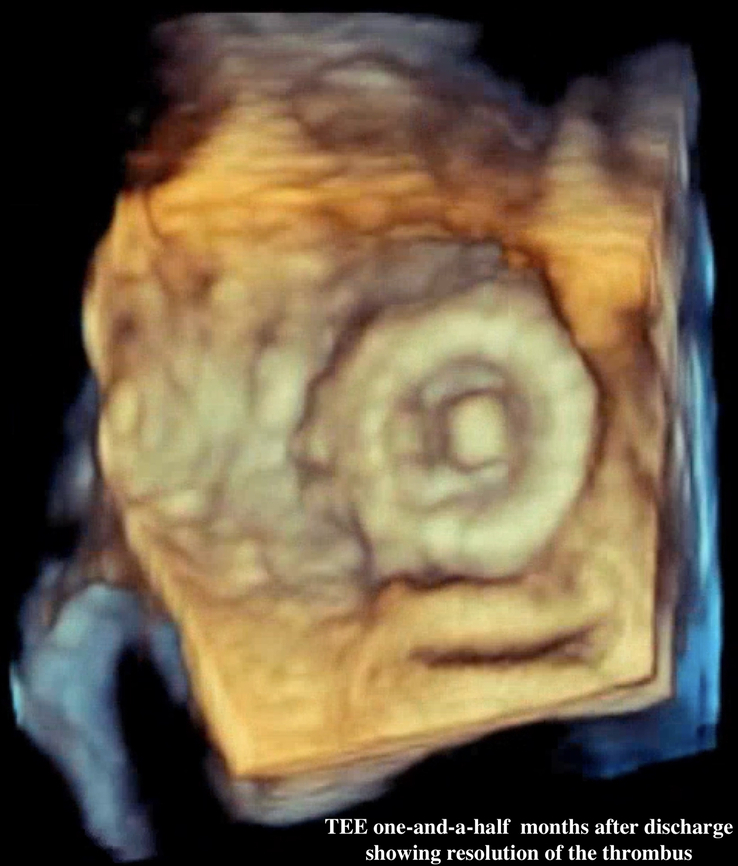
Transesophageal echocardiography image obtained 1.5 months after discharge showing thrombus resolution.

About one-and-a-half months later, the patient underwent a TEE, which showed a well-seated LAAO device, within the left atrial appendage, and no thrombus or peridevice leak noted (Fig. 3). Warfarin was still continued given the patient’s recent history of thrombus while on uninterrupted oral anticoagulation with apixaban, with a tentative plan for an electrophysiology study with AF ablation. The patient’s perspective on his course of events was that of curiosity and confusion regarding how he developed a thrombus, despite being adherent to his anticoagulation regimen, along with an eagerness to resolve his AF.

## Discussion

The etiologies of AF are multifactorial with increasing age, male sex, structural cardiovascular changes, sedentary lifestyle, obesity, diabetes mellitus, hypertension, obstructive sleep apnea, and smoking all contributing^1^. Abnormal electrical activity propagated from the ectopic focus within the atria causes the heart to beat in an irregularly irregular rhythm in which the blood becomes turbulent, increasing the likelihood of thrombus formation^1^. It is estimated that the risk of stroke in AF patients is five times higher than in those without AF^1^. The use of validated scoring criteria, such as CHA2 DS2–VASc, assists in stroke risk stratification amongst AF patients, with oral anticoagulants and nonpharmacologic procedural interventions, such as LAAOs, utilized in mitigating stroke risk^1^.

DCCV has been associated with significant changes to the physiological dynamics of the myocardium. The passage of energy during DCCV has been noted to incite myocardial injury, manifested as transient ST elevations on electrocardiograms, with cellular damage, oxidative stress, fibrosis, and inflammation contributing^6^. Statistically significant elevations in high-sensitivity troponin (HST) biomarkers were noted in a retrospective study amongst AF patients undergoing DCCV regardless of the shock energy administered^7^. Yet, the clinical significance was unclear as the increase was minimal, suggesting that other causes of myocardial injury should be explored^7^. DCCV has also been noted to further propagate myocardial edema and incite fibrosis in the long-term setting, particularly in patients at an increased risk of myocardial injury prior to cardioversion^8^. Further studies are needed to evaluate the impact of myocardial injury after DCCV with respect to patient outcomes.

The need for TEE prior to cardioversion in patients with LAAO devices remains unclear given current guidelines. The decision to obtain a TEE before repeat cardioversion in our patient, despite current guidelines stating it was not explicitly necessary, reflects the ethical challenges in electrophysiologic procedures^9^. Electrophysiologists must often balance adhering to decision-making processes while ensuring that the principle of beneficence is not jeopardized^9^. In hospital settings, the decision to obtain a TEE before cardioverting patients with LAAO devices is at the discretion of the cardiologist performing the procedure, thus highlighting the obligation of physicians to alleviate the risk of harm in the absence of uniform criteria associated with ethical approaches in cardiac electrophysiology^4,9^.

While LAAO devices prevent thromboembolism formation in the left atrial appendage, there remains a risk of thromboembolism postdevice implantation since thrombus formation may occur on the device itself^10^. This phenomenon, known as a device-related thrombus (DRT), occurs due to incomplete endothelialization of the LAAO device, promoting a thrombogenic environment and ultimately an increased risk of embolization^10^. It is estimated that LAAO patients with a device-related thrombus have more than three times an increased risk of stroke and systemic embolism than the general population, with risk factors such as vascular disease, prior stroke, increased diameter of the left atrial appendage, and decreased left ventricular ejection fraction contributing^10^. Thus, it is suggested that a rigorous surveillance protocol, through routine TEEs, be considered in these patients when risk factors for DRT are evident^10^. The use of TEE prior to cardioversion in patients with LAAO devices can help localize the presence of DRT and subsequent therapy with oral anticoagulation being needed prior to cardioversion^4^.

The detection of a left atrial thrombus in our patient who was adherent to apixaban, challenges current guidelines that recommend proceeding with cardioversion without the need for TEE in AF patients compliant with at least 3 weeks of anticoagulation^2,3^. Previous studies have shown that the incidence of new-onset left atrial thrombus prior to DCCV amongst patients with a recently unremarkable TEE was not uncommon and individuals with increased risk factors for thromboembolism should still be reevaluated with TEE^11,12^. There remains a risk of stroke after DCCV due to atrial stunning, a transitory dysfunction of the left atrial appendage and left atria, promoting stasis development and increasing the risk of thromboembolism^13,14^. The duration of AF itself along with the severity of both left atrial dilation and left ventricular systolic dysfunction are suggested to be contributing risk factors of atrial stunning^15^.

Our case report has several strengths and limitations. One strength is that our study serves as a platform to bring awareness, challenge, and explore current guidelines regarding the need for TEE in AF patients compliant with anticoagulation for a minimum of 3 weeks^2,3^. Other strengths include the educational benefit in highlighting the rarity of device-related thrombus, despite anticoagulation, while emphasizing the pharmacovigilance of anticoagulation itself. Limitations include the absence of a control group and the verifiability of whether our patient was truly compliant with his anticoagulation regimen. The fact that our patient developed a thrombus, despite anticoagulation, does not necessarily imply the generalizability that all AF patients should undergo TEE before cardioversion. Rather, given that the functional impairment of atrial stunning can be appreciated by assessing for left atrial appendage emptying velocity through TEE, further studies are needed to evaluate the benefit of routine TEE in recently cardioverted patients with LAAO devices who possess increased risk factors for stroke^13–15^.

## Conclusion

While the use of LAAO devices and anticoagulation serve to decrease the risk of thrombus formation and thromboembolism, it remains unclear how said risks are impacted by atrial stunning, especially in patients with increased risk factors for stroke. Current guidelines are unclear regarding the need for TEE both prior to and after cardioverting AF patients with LAAO devices on anticoagulation, with further studies indicated to evaluate the need for routine TEE in these patients.

## Ethical approval

Ethics clearance was not necessary as this was a case report. There was no research study conducted and Institutional Review Board approval was not needed.

## Consent

Written informed consent was obtained from the patient for publication of this case report and accompanying images. A copy of the written consent is available for review by the Editor-in-Chief of this journal on request.

## Sources of funding

We, the authors, have nothing to declare in this category as it is not applicable.

## Author contributions

H.A., M.I., A.P., M.H., T.P., O.K., and J.V.: conceptualization; H.A., M.I., A.P., M.H., and T.P.: writing – original draft; H.A., M.I., A.P., M.H., T.P., O.K., and J.V.: writing – review and editing; H.A., M.I., A.P., M.H., T.P., and O.K.: investigation; O.K and J.V.: supervision.

## Conflicts of interest disclosure

We, the authors, have no conflicts or potential conflicts of interest to disclose.

## Research registration unique identifying number (UIN)

We, the authors, have nothing to declare in this category as it is not applicable to our case report.

## Guarantor

Hasaan Ahmed, MD is the guarantor who accepts full responsibility for the case report and controlled the decision to publish.

## Data availability statement

We, the authors, have nothing to declare in this category as it is not applicable.

## Provenance and peer review

Our paper was not invited for publication.
